# Validation of baking as a kill-step for controlling Shiga toxin-producing *Escherichia coli* during traditional crust pizza baking process

**DOI:** 10.3389/fmicb.2022.1001597

**Published:** 2022-10-06

**Authors:** Arshdeep Singh, Lakshmikantha H. Channaiah

**Affiliations:** Division of Food, Nutrition & Exercise Sciences, College of Agriculture, Food and Natural Resources, University of Missouri, Columbia, MO, United States

**Keywords:** pizza, pepperoni, thermal inactivation, STEC, validation, *E. coli*

## Abstract

A study was conducted to validate a simulated traditional crust pepperoni pizza baking process to control Shiga toxin-producing *Escherichia coli* (STEC) and to determine the heat resistance characteristics of STEC in pizza dough. Pizza dough and pepperoni slices were inoculated with 7 strains STEC cocktail and baked at 500°F (260°C) for 12 min using a conventional kitchen oven followed by 15 min of ambient air cooling. The mean internal temperature of the pizza reached 209.32 ± 1.94°F by the end of 12 min of baking and dropped to 137.90 ± 2.88°F after the 15 min ambient air cooling. The a_w_ and pH of the traditional crust pizza did not alter significantly during the baking process. The STEC population decreased by >5 log CFU/g in traditional crust pizza after 12 min of baking. Where pepperoni slices were used as a source of STEC introduction, a reduction of >6.5 log CFU/g was observed. The *D*-values of STEC cocktail in pizza dough at 55, 58, 61°C were 49.5 ± 4.10, 15.3 ± 0.68, and 2.8 ± 0.31 min, respectively. The *z*-value of STEC was 4.8 ± 0.16°C. This study validated that a typical traditional crust pizza baking process with ~209°F internal temperature for at least 12 min will result in 5 log reductions in STEC population.

## Highlights

- A first scientific validation study demonstrating pizza baking process to control STEC.
- The STEC population decreased by >5 log CFU/g in traditional crust pizza during the 12 min of baking.
- The data will be useful for bakers in optimizing the pizza baking process and to achieve food safety.

## Introduction

Foodborne illness continues to be a huge burden for the United States, with an estimated 47.8 million illnesses, 127,839 hospitalizations and 3,037 deaths annually (CDC, 2018). Some of the important pathogens that cause foodborne illness in the U.S. include norovirus, *Salmonella*, *Clostridium perfringens*, *Campylobacter* spp., Shiga toxin-producing *Escherichia coli* (STEC), and *Listeria monocytogenes* (CDC, 2018; Dhaliwal et al., 2021). Among the variety of food products, foods with low water activity are generally considered to be less susceptible to pathogenic microbial contamination. However, in recent years, low water activity food products and ingredients are increasingly implicated in food-borne disease outbreaks due to the presence of STEC and other pathogenic bacteria (Beuchat et al., 2013; Crowe et al., 2017). Due to its low-water activity, wheat flour was traditionally regarded as relatively safe ingredient by food industry experts. However, it has received increased attention in recent years due to the presence of pathogenic *E. coli* and has been responsible for food product recalls and foodborne illness outbreaks (CDC, 2009, 2021, 2022a; Crowe et al., 2017; Gieraltowski et al., 2017; Harris and Yada, 2019; FDA, 2021; Tack et al., 2021). For instance, in 2009, the Center for Disease Control (CDC) reported a multistate outbreak of *E. coli* O157:H7 infections in the U.S. due to eating raw refrigerated cookie dough sickening 72 people in 30 states (CDC, 2009). In 2016, another multi-state outbreak of STEC infections was linked to flour sickening 63 people in 24 states (FDA, 2017). FDA’s traceback investigation team identified flour as the source of this outbreak in which 17 sick people were hospitalized and one patient developed the hemolytic uremic syndrome. The FDA tested flour samples and detected strains of *E. coli* O121 and O26, resulting in a massive flour recall (CDC, 2016; FDA, 2017). The CDC identified that people got sick either by eating or by handling raw dough (FDA, 2017; CDC, 2022b). In another multi-province outbreak incident, 29 cases of STEC O121 were reported in Canada between 2016 and 2017 due to consumption of raw flour (Morton et al., 2017). In 2019, a major milling company in the U.S. recalled ~600,000 pounds of flour due to the presence of *E. coli* O26 which was discovered during sampling of the five-pound finished product (FDA, 2019). In May 2022, frozen pizzas caused a foodborne illness outbreak causing 2 deaths and 53 illnesses in 12 regions of metropolitan France (Marler, 2022). Initial investigation by Public Health France linked these illnesses to STEC O26 and STEC O103 strains. Raw wheat flour is widely used in the preparation of staple foods, and a wide variety of bakery food products manufactured in North America and around the world. Additionally, the wheat supply chain plays a significant role in the country’s economy. For example, with ~800,000 employees, U.S. retail and commercial bakeries generate $3 and $31 billion annual revenues, respectively, (Woodruff, 2019). It is therefore essential to strengthen food safety parameters, as flour has a long shelf life and pathogenic bacterial cells can survive through prolonged storage.

Besides raw flour, pathogens such as STEC can be introduced into bakery and other food products through a wide range of ingredients such as egg, milk products, coconut, peanut butter, fruits, spices, employees, and other environmental factors (USDA, 2012; Forghani et al., 2018; Channaiah et al., 2019; FDA, 2022). Although STEC cannot grow in raw flour due to its low water activity, previous studies have shown that STEC can survive for several weeks, months and up to 2 years (Forghani et al., 2019; Gill et al., 2020; Michael et al., 2022). Pathogenic bacterial growth could occur when water is introduced during the preparation of batter or other food products. The presence of pathogens such as STEC in finished food products such as pizza could create a public health risk if thermal processing steps such as baking is not adequate. Therefore, it is critical that food manufacturers conduct a comprehensive risk assessment to identify potential hazards that may be associated with each ingredient and implement an effective thermal processing step as part of the food safety plan. Additionally, it is important to validate thermal food process preventive control steps such as baking, cooking, roasting, boiling, etc. to ensure food safety and to comply with U.S. FDA’s Food Safety Modernization Act’s (FSMA) validation (21 CFR § 117.160) requirement (FDA, 2018a). The FSMA act implemented in 2011, mandates that the facilities subject to the preventive control requirements must validate their kill steps or food process preventive controls as appropriate to the nature of the preventive control and its role in the facility’s food safety system (Kennedy et al., 2014; FDA, 2018a,b). To our knowledge, there have been no prior studies on thermal inactivation and heat resistance characteristics of STEC in pizzas. Therefore, a study was conducted to validate the effectiveness of baking as a kill-step for controlling potential STEC contamination during pizza manufacturing. We choose pizza as it is one of the most popular and most widely consumed savory bakery products in North America, Europe, and Australia, with sales of over $37 billion annually just in the U.S. alone (Bakerpedia, 2017). STEC was selected as the main pathogen of concern as it can survive in raw flour for extended periods, and because of its role in flour recalls and foodborne illness outbreaks. The specific research objectives are to validate a simulated baking (500°F for 12 min) of inoculated traditional crust pizza to control STEC and determine the thermal inactivation kinetic parameters (*D*- and *z*-values) of STEC in traditional crust pizza dough.

## Materials and methods

### Experimental design

Two separate studies were conducted to validate the traditional crust pizza baking process to control STEC contamination and determine the heat resistance characteristics (*D-* and *z-*values) of STEC in pizza dough. Additionally, the water activity (a_w_), humidity, and pH of the product were determined during the baking process. The baking validation study was designed as a randomized complete block (replications as block) with 11 treatments (dough +10 times) and one oven temperature: dough, 0-min, 2-min, 4-min, 6-min, 8-min, 10-min, 12-min, and at 5 min intervals during 15-min of ambient temperature cooling followed by baking (B + C). Analysis of variance for the surviving STEC populations (log CFU/g), a_w_, pH and humidity were analyzed using Minitab® 19 statistical software (Minitab, LLC, State College, PA). Three independent replications were conducted for STEC, and all the experiments were conducted in duplicates.

For the *D*- and *z*-value determination, the study was designed as a randomized complete block, with replications as blocks. The *D*- and *z*- values of STEC cocktail in pizza dough were carried out using three target temperatures. Linear regression graphs were plotted using Microsoft^®^ Excel 2021 (Microsoft Corp., Redmond, WA), and statistical differences in *D*- and *z*-values were determined using one-way ANOVA using Minitab® 19 statistical software.

### STEC strains used in this study

In this study, we used seven strains of STEC cocktail culture. Two strains of *E. coli* O157:H7 and one strain of *E. coli* O26 were obtained from the American Type Culture Collection (ATCC®; Manassas, VA). Additionally, two strains each of *E. coli* O157:H7 and *E. coli* O121 from the University of Missouri (Columbia, MO, United States) culture collection were used in this study. Since a wide range of STEC serotypes were detected in flour and flour-based food products, care was taken to pool seven prominent STEC strains based on human clinical significance, product recalls and associated foodborne illness outbreaks. Please refer Table 1 for details of STEC strains used in this investigation. All the cultures used in this study were grown in Brain Heart Infusion (BHI; Difco™, Becton, Dickinson and Company, Franklin Lakes, NJ) broth and stored at 4°C.

**Table 1:** Shiga toxin producing *E. coli* strains used in this study.

STEC	Strain	Origin
*E. coli* O157:H7	ATCC 43894	Hemorrhagic colitis outbreak
*E. coli* O157:H7	ATCC 43895	Feces from outbreak of hemorrhagic
*E. coli* O26	ATCC BAA2196	Stool samples
*E. coli* O157:H7	C7927	Patient (apple cider, CDC)
*E. coli* O157:H7	MF-1847	Beef (USDA)
*E. coli* O121	PT-914 (UMCC)	Unknown
*E. coli* O121	DA1 (UMCC)	Human

ATCC®: American Type Culture Collection (Manassas, VA), CDC: The Centers for Disease Control and Prevention, USDA: United States Department of Agriculture, UMCC: University of Missouri Culture Collection.

### Inoculum preparation

All STEC cultures were individually activated by transferring 0.1 ml of stock cultures into 10 ml of BHI broth and incubated at 37°C for 24 h. After the incubation, individual working cultures were obtained for each experiment by transferring 0.1 ml of cultures into 45 ml of BHI broth in 50-ml centrifuge tubes and incubated at 37°C for 24 h. After the incubation, six 50-ml centrifuge tubes containing six individual STEC cultures were centrifuged at 6,000 rpm for 15 min to harvest pallet of bacterial cells (Michael et al., 2022). The harvested STEC pallet was then resuspended using five ml of sterile water and mixed. The resuspended individual bacterial solutions were then mixed in equal proportions to obtain a 7-strain cocktail of STEC and used as master inoculum for inoculating the flour. The initial STEC population in the master inoculum was 10.20 ± 0.08 log CFU/ml.

### Inoculation of flour

Since flour is the principal ingredient, it was used as a source of STEC contamination in this study. The flour inoculation with STEC was carried out according to the method described by Channaiah et al. (2016). For each replication, bread flour (250 g) was weighed and evenly spread in a sanitized sealable plastic tub (~38 × 26 × 7 cm, Rubbermaid Inc., Huntersville, NC, United States). A spray nozzle attached to a 50 ml centrifuge tube containing master inoculum was calibrated to spray 1 ml of master inoculum per squirt. The flour tub was placed inside a Class II, Type A2 Biological Safety Cabinet (LabConco Corporation, Kansas City, MO) and mist inoculated (8 ml) using master inoculum. The inoculated flour was thoroughly mixed inside the plastic container and dried back to original pre-inoculation weight by incubating for ~6 h at 37° with open lids (Unger et al., 2021). Inoculated flour was thoroughly mixed using a sanitized spatula, sealed in airtight plastic containers, and stored at room temperature (~21°C) until used within 2 days.

### Inoculation of pepperoni slices

A separate study was carried out to evaluate the pizza baking process on STEC survival when introduced through pepperoni slices. For each replication, 60 grams of pepperoni were weighed in a sanitized sealable plastic tub (~38 × 26 × 7 cm, Rubbermaid Inc., Huntersville, NC, United States), transferred inside a biosafety cabinet, and mist inoculated with STEC cocktail culture using a spray nozzle as described earlier. Each side of the pepperoni slices was mist inoculated with ~4 ml of master inoculum. Following inoculation on one side, a three-minute interval was provided to facilitate STEC adherence, later the slices were flipped using a sterile spatula and inoculated on the other side. The inoculated pepperoni slices were then dried back to the original pre-inoculation weight by incubating for ~2 h at 37°C with open lids and used on the same day.

### Preparation of pizza dough

All the activities involving inoculated pizza dough preparation were carried out in a biosafety cabinet. All the ingredients required to make traditional crust pepperoni pizza were purchased from a local grocery store in Columbia, MO, United States. The traditional crust medium sized pepperoni pizza baking (slow rise dough) recipe was used in this study. The traditional crust pepperoni pizza recipe, baking parameters and technical assistance were given by the American Institute of Baking International (AIB International, Manhattan, KS, United States). In a mixing bowl, dry yeast (Fleischmann’s Active dry yeast, ACH Food Companies, Inc., Chicago, IL, United States) was added to a sugar-water solution and left for 10 min for yeast activation. Subsequently, salt, olive oil and inoculated flour were added to the mixing bowl attached to a kitchen mixer (Classic KitchenAid®, Benton Harbor, MI) and mixed for 2 min at speed 1. Please refer to the Table 2 for traditional crust pepperoni pizza recipe. Later, the prepared dough was placed in a 37°C incubator for 1 h for proofing. After 1 h, the dough was stretched and rolled using a sanitized classic wood rolling pin to make a 12″ diameter pizza base with ~0.5 cm thickness. The pizza base was placed on a 16″ diameter pizza pan (Wilton Industries, Inc., Woodridge, IL), and the traditional pizza sauce was spread onto the pizza base, followed by shredded mozzarella cheese (Kraft Heinz, Chicago, IL, United States) and inoculated pepperoni slice toppings (Hormel Foods Corporation, Hormel Place, Austin, MN, United States).

**Table 2:** Traditional crust pepperoni pizza – recipe.

Ingredient	Weight (*g*)
Sugar	4.5
Active yeast	1.75
Salt	3.0
Olive oil	1.5
Inoculated flour	200
Cooking oil for spray	2 squirts
Pizza sauce	70
Mozzarella cheese	150
Pepperoni	45
Water	140 ml

### Pizza baking

**Figure 1:**
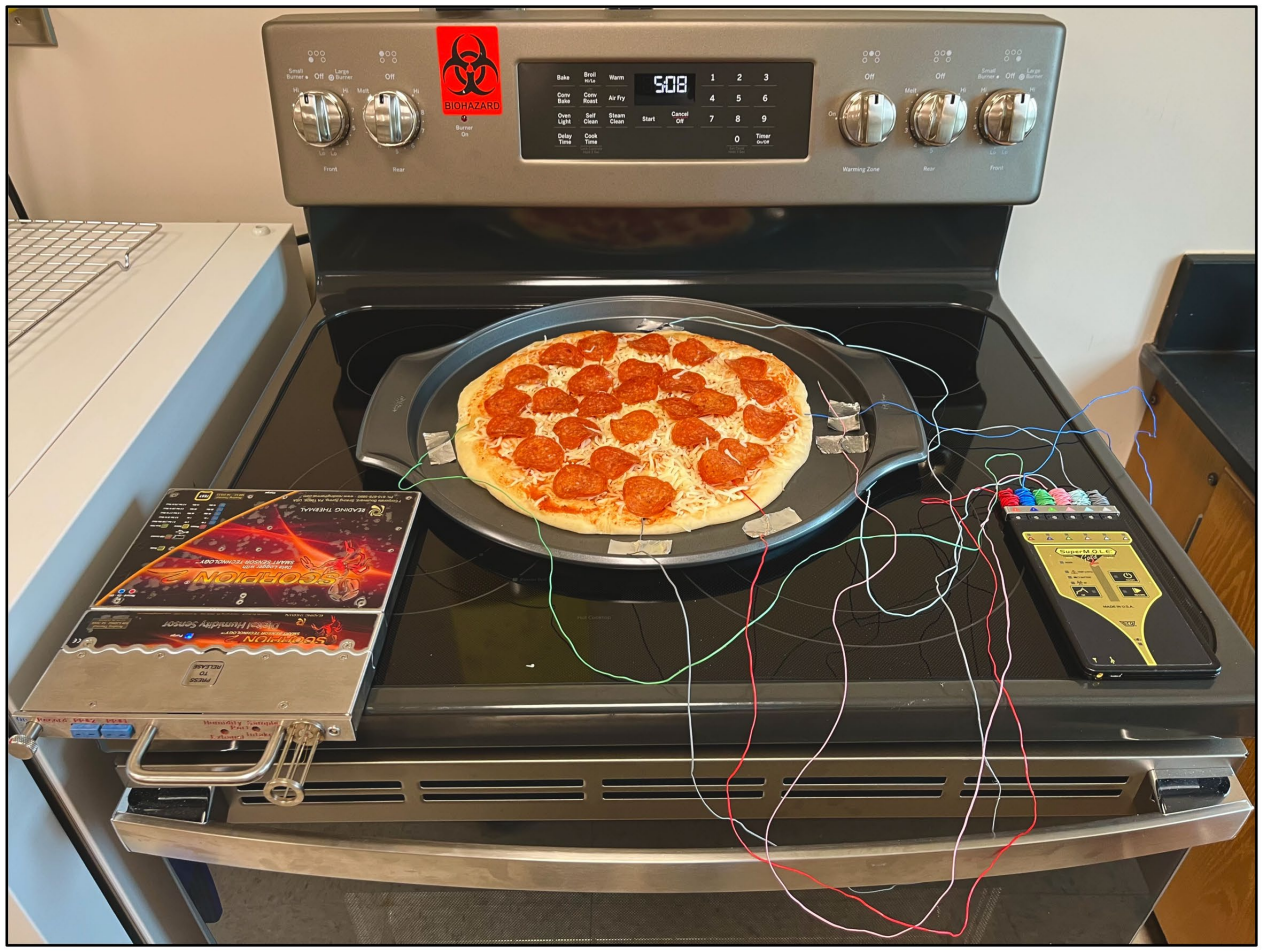
Pizza baking validation study setup.

Before executing the STEC cocktail inoculated pizza baking validation study, a series of preliminary trials were carried out at different bake time and temperature combinations to optimize the traditional crust pizza baking process using the kitchen oven. Expert baking professionals from AIB International were consulted to establish the optimum baking parameters to mimic the commercial end use quality parameters using a conventional kitchen oven [GE Oven, (Model #JBS86SPSS), Louisville, KY, United States]. After a series of trials, it was determined that baking at 500°F for 12 min is an optimal commercial baking setting. Before baking the pizza, the kitchen oven was preheated to 500°F for at least 1 h and the temperature was confirmed using a digital thermometer (Fluke 51–2 Thermometer, Everett, WA, Unioted States), connected to an 80TK thermocouple. A 6-channel data logging system (SuperM.O.L.E.^®^ Gold 2, ECD, Milwaukie, OR) with six K-type thermocouples were used to monitor the temperature in pizza and the oven every second throughout the baking. Prior to starting the pizza baking process, four thermocouples were inserted (at the geometric center 3″ away from the edge) on all four sides of the pizza at equal distance from each other and another thermocouple was inserted at the center of the pizza. Please refer to Figure 1 for details. All the thermocouples were carefully inserted into the geometric center (0.5 cm thick pizza base) to record the internal temperature (the cold spot) of the pizza representing each side including the center. A thermocouple was also affixed to the side of the pan to monitor the oven air temperature during the pizza baking process. Before placing the pizza pan into the oven, the thermocouples were logged at 1-s intervals over the defined baking and cooling (B + C) periods. The stopwatch was started as soon as the pizza base was placed inside the oven, and pizza samples (25 g) were sampled every 2 min using a T-handle core sampler. After 12 min of baking, pizza was removed from the oven and allowed to cool at room temperatures for 15 min. For the sampling, the oven door was opened for less than 10 s to collect the pizza samples during baking.

### Water activity (a_w_), pH and humidity

The a_w_ of the pizza samples were determined at 25°C, using an Novasina Labswift Portable water activity meter (Novasina-AG, Lachen, Switzerland) as described by Michael et al. (2020). The a_w_ and pH for baking validation study were measured during baking (dough, 0, 2, 4, 6, 8, 10, 12 min) and at 17, 22, and 27 min during ambient cooling. At each sampling point, for a_w_, the samples were placed in Novasina sample cups (diameter 40 mm × Height 13 mm) with lids (Novasina-AG, Lachen, Switzerland) within 10 s of removal from the oven, and analyzed within 30 min. For pH, a 15 g of pizza sample was added to 45 ml of deionized water, and the mixture was stirred continuously until a stable pH reading was attained using a pH meter (FiveEasy F20 pH meter, Mettler-Toledo, Greifensee, Switzerland). A separate triplicate study was carried out to measure the humidity of the thermal environment inside the oven during baking using the Scorpion^®^ 2 Digital Humidity Sensor (Reading thermal, Sinking Spring, PA, United States). Before starting the pizza baking process, the humidity sensor enclosed in the thermal barrier was preheated to ∼65°C and placed inside the oven to record the humidity ratio (kg of moisture / kg of dry air). The humidity ratio was recorded every second during the pizza baking process.

### *D*- and *z*-values determination

The pizza dough prepared using inoculated flour (seven-serovar STEC cocktail) to achieve a target population of ~6 log CFU/g. The *D*-values were determined at 55, 58, and 61°C temperatures using the methodology described by Channaiah et al. (2018). For this we used a temperature-controlled water bath (Precision CIR19, Thermo Fisher Scientific, Newington, NH, United States) and sanitized aluminum thermal-death-time (TDT) disks (Engineering Shop, Washington State University, Pullman, WA, United States). The internal height and diameter of TDT disks were 5 and 50 mm, respectively. Ten grams of pizza dough prepared from the inoculated flour was individually transferred into four regular and one T-type thermocouple connected with TDT disks. All five TDT disks were transferred to preset hot water baths at specified (55, 58 and 61°C) temperatures. Once the dough reached the target temperature inside the TDT disks, the first TDT disk was quickly removed and placed in an ice water until further processing. The remaining TDT disks were then randomly removed from the hot-water bath at pre-determined specified temperatures, and the TDT disk connected to the thermocouple removed at the last. Two digital thermometers (Fluke 51–2 Thermometer, Everett, WA), connected to the thermocouples were used to monitor the temperature of water and the dough in the TDT disk, respectively.

### Microbial analysis

The microbial analysis was carried out according to the method described by Channaiah et al. (2016). Duplicate samples (15 grams) of inoculated post proof dough and pizza samples were collected at 0-, 2-, 4-, 6-, 8-, 10-, and 12-min during baking and samples collected during ambient cooling were diluted in 45 ml of chilled peptone water buffer. The samples were stomached for 1 min using a stomacher (Stomacher® 400 Circulator, Worthing, West Sussex, United Kingdom) and stored at 4°C until analyzed within 30 min. Serial dilutions of each sample were spread plated in duplicates on injury-recovery media to quantify both healthy and sub-lethally injured STEC populations (Channaiah et al., 2016). The BHI agar plates were incubated at 37° for 4 h, then overlaid with a selective agar (MacConkey agar) and further incubated for 20 h (Gill et al., 2014).

For *D*- and *z*-values determination, the pizza dough samples (10 g) were transferred into stomacher bags containing 10 ml of 0.1% peptone water, stomached for 2 min, stored for 1 min at refrigeration temperature, and analyzed within 30 min (Channaiah et al., 2016). The BHI plates were incubated at 37°C for 4 h and then overlaid with the selective MacConkey agar. After the incubation, the microbial colonies were enumerated using the Reichert Quebec Darkfield Digital Colony Counter-110 V (Buffalo, New York, United States) to determine the concentration of survived STEC cells in pizza samples (pre and post baking) and expressed as Colony Forming Units (CFU)/g.

## Results

**Figure 2:**
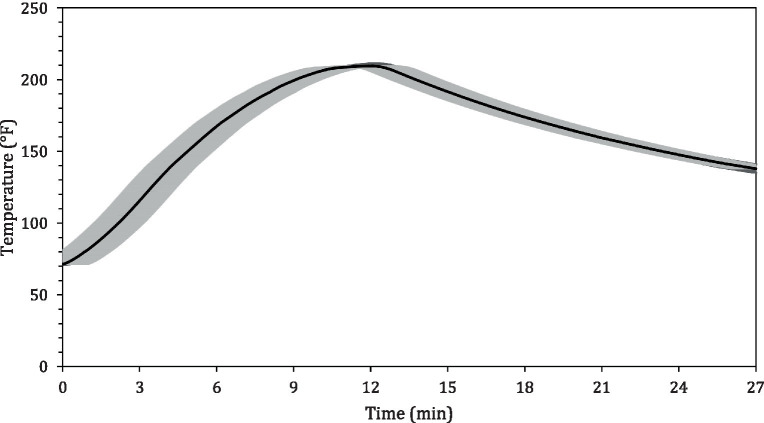
Mean (± SE; *n* = 5) internal temperature profile of pizza during baking at 500°F for 12 min followed by 15 min of ambient air cooling.

**Figure 3:**
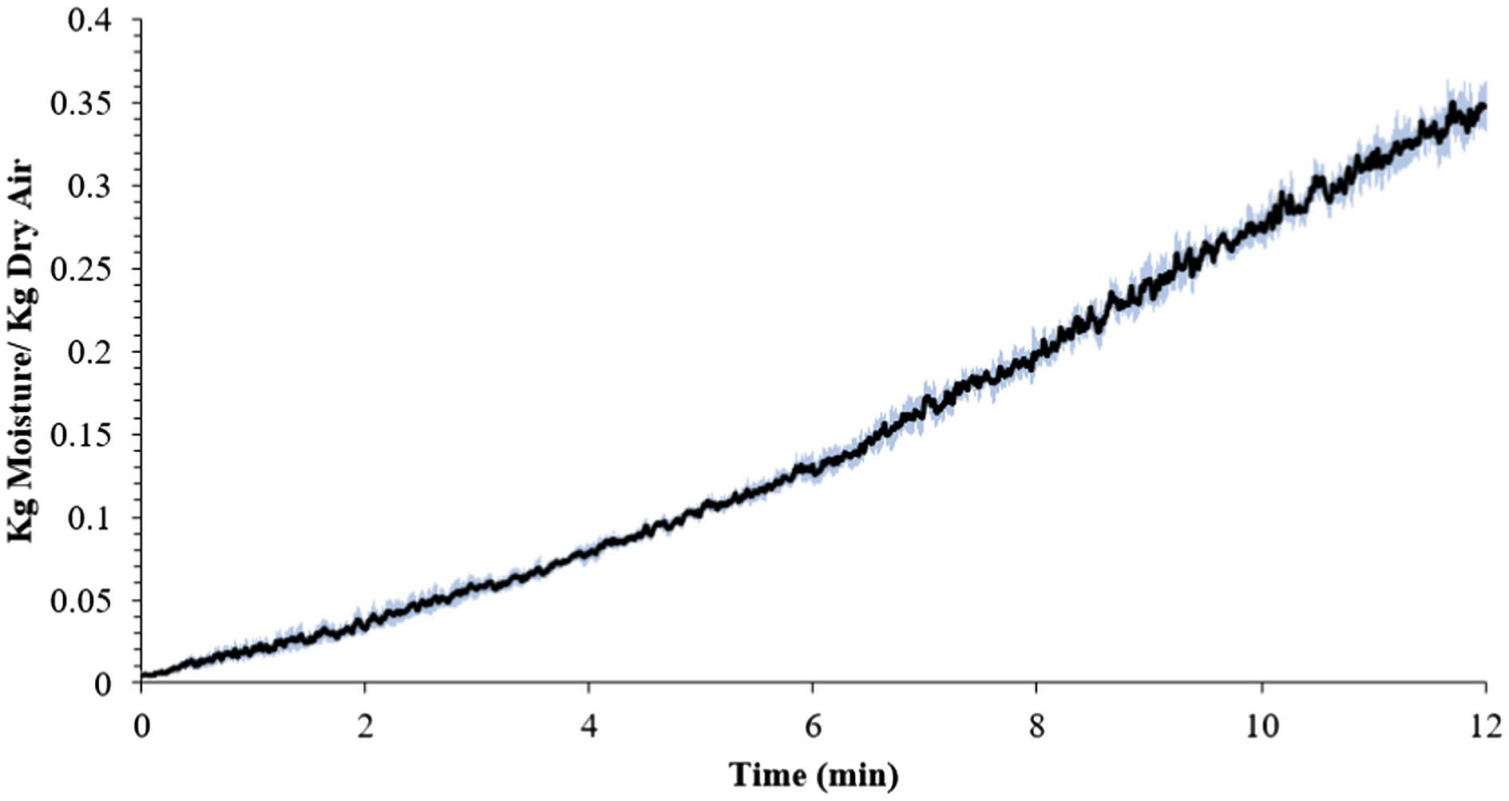
Mean humidity ratio (kg moisture/kg dry air) (± SE) of oven during 12 min of baking at 500°F oven temperature.

**Figure 4:**
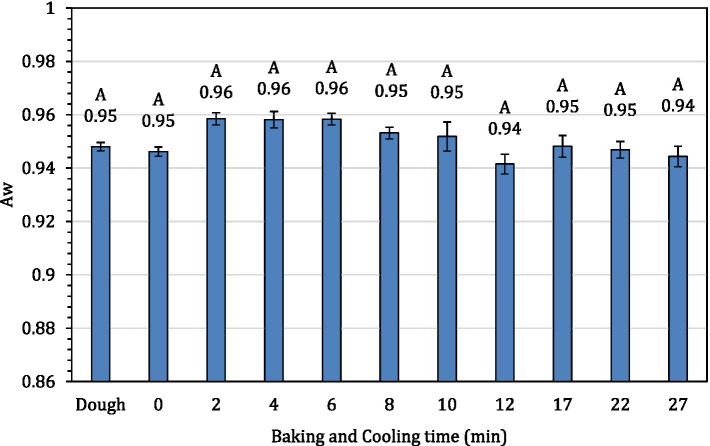
The water activity of pizza samples during 12 min of baking followed by 15 min of ambient cooling.

**Figure 5:**
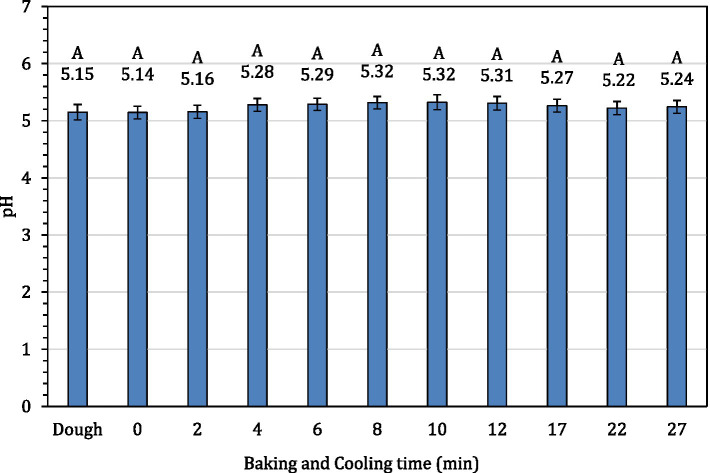
pH of pizza samples during 12 min of baking followed by 15 min of ambient cooling.

The average internal temperature of the traditional crust pizza during the baking process is shown in Figure 2. The average internal temperature of the pizza after 12 min of baking and at the end of 15 min of ambient air cooling was recorded as 209.32 ± 1.94°F and 137.90 ± 2.88°F, respectively. As shown in Figure 3, the humidity ratio of the oven at the start and the end of the baking was 0.0045 ± 0.0021 kg moisture/kg dry air and 0.3477 ± 0.0154 kg moisture/kg dry air, respectively. Figures 4, 5 represent the a_w_ and pH of the pizza samples throughout the baking and cooling process, respectively. The a_w_ ranged from 0.946 ± 0.001 to 0.9583 ± 0.0021, whereas the pH ranged from 5.14 ± 0.11 to 5.32 ± 0.13 during baking followed by 15 min of ambient air cooling. The post proof dough had an a_w_ of 0.948 ± 0.001 and a pH of 5.14 ± 0.13. In general, the a_w_ and pH of the traditional crust pizza did not change significantly during the baking process.

**Figure 6:**
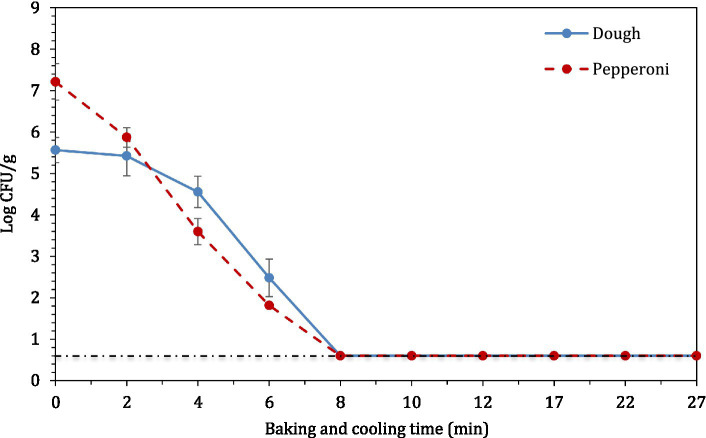
The log_10_ reduction in STEC population in pizza dough or crust and pepperoni slices during 12 min of baking followed by 15 min of ambient cooling.

The concentration of STEC (7 strain STEC cocktail) master inoculum used to inoculate raw wheat flour was ~10 log CFU/ml. The dried, inoculated wheat flour retained 5.46 ± 0.10 log CFU/g STEC population. In the post proof dough, the population of STEC was 6.45 ± 0.17 log CFU/g. However, the STEC population decreased by >5 log CFU/g after 12 min of baking (Figure 6) where microbial populations decreased below the enumeration detection limit (0.6 log CFU/g). When pepperoni slices were used as a source of STEC introduction, there was a reduction of >6.5 log CFU/g. The initial populations of STEC on pepperoni slices were 8.95 ± 0.19 log CFU/g.

**Figure 7:**
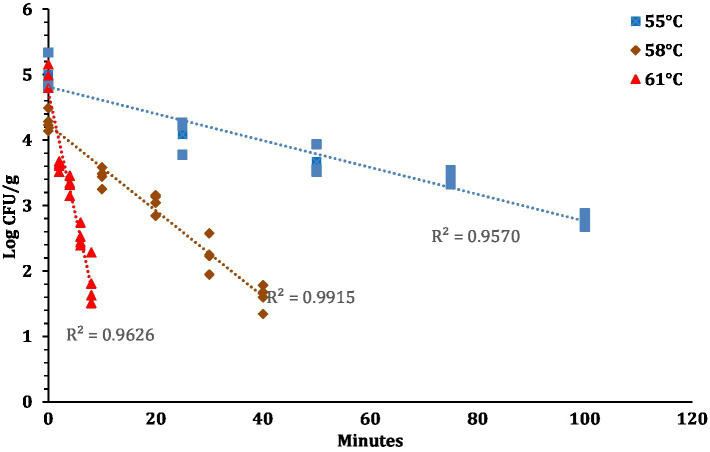
STEC population (Log CFU/g; mean and three replications) versus time (minutes) graphs used for calculating *D*-values at 57, 58, and 61°C.

**Figure 8:**
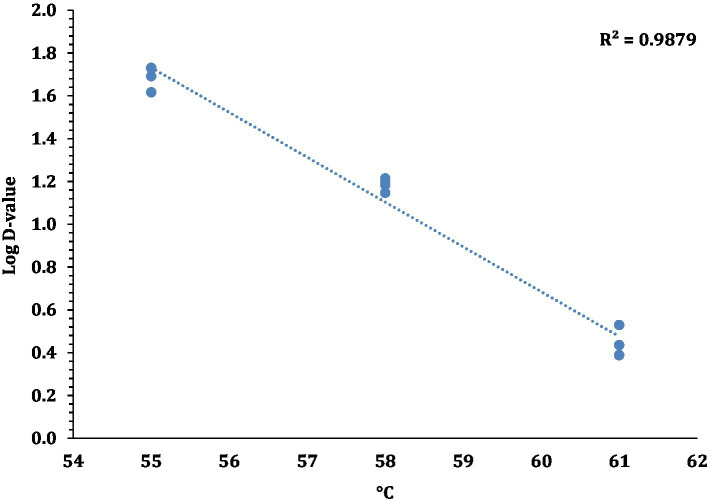
Log *D*-values (mean and three replications) versus temperature (°C) graphs used for calculating *z*-values for STEC.

The linear regression plot (log STEC population vs. time) was constructed to calculate the *D*-values of the 7-strain cocktail of STEC in traditional crust pizza dough. The calculated *D* values for STEC at 55, 58, and 61°C as shown in Figure 7 were 49.5 ± 4.10, 15.3 ± 0.68, and 2.8 ± 0.31 min, respectively. The *z*-values of STEC (4.8 ± 0.16°C) were calculated by plotting a thermal death time curve using the log *D*-values versus respective exposed temperature graphs presented in Figure 8. The *D*- and *z*-values of the 7-strain STEC are presented in Table 3.

**Table 3:** Mean (± SE; *n* = 3) *D*-values (min) and *z*-values (°C) of STEC.

Item	Shigella toxin-producing *E. coli*
55.0°C	49.5 ± 4.10
58.0°C	15.3 ± 0.68
61.0°C	2.8 ± 0.31
*z*-value	4.8 ± 0.16

## Discussion

It is important to note that wheat flour is a raw agricultural product that has not been processed to control pathogenic bacteria that cause food-borne illnesses. Although there is some reduction, the flour milling process is not designed to control microbial contamination coming from the fields, during storage at grain elevators and transportation. Heat treatment steps such as baking, cooking, roasting, etc. are generally applied by the downstream processors (e.g., bakery, food manufacturers) to ensure the safety of the final food product.

The average temperature of traditional crust pizzas in this study increased steadily over 12 min of baking and dropped significantly during ambient air cooling. The oven door was opened to collect pizza samples (<10 s each time) during the 12-min baking. Unger et al. (2021) validated brownie baking step for controlling *Salmonella* and *Listeria monocytogenes*. The authors concluded that opening the oven door for <10 s does not affect the product temperature significantly but does impact the oven air temperature for a very short period of time. However, it regained its previous temperature levels once the oven door was closed. As predicted, the overall humidity level of the oven increased steadily during the pizza baking process (Figure 3). This was expected due to the production of steam from the moisture inside the pizza dough and pepperoni toppings. Previously published results (Channaiah et al. (2019) reported an increase in overall humidity level from 0.050 ± 0.0009 to 0.261 ± 0.0158 kg moisture/kg dry air during 35 min of whole wheat multigrain bread baking process. The change in humidity pattern observed in this study is in line with Channaiah et al. (2019).

The a_w_ and pH of the traditional crust pizza remained almost constant throughout the baking and cooling process. The chemical composition, pH and a_w_ of variety of pizzas can vary based on ingredients and baking parameters used. The a_w_ in our study ranged from 0.946 ± 0.001 to 0.9583 ± 0.0021. These findings are in line with data reported by previous investigators. Singh et al. (2012) observed a_w_ of baked pizza during shelf-life extension of fresh ready-to-bake pizza by the application of modified atmosphere packaging and reported a_w_ ranging from 0.975 in unbaked pizza samples to 0.963 in packed baked pizza. El-Beltagi et al. (2017) studied the impact of using chickpea flour and dried carp fish powder on pizza quality and reported a water activity of 0.987 in 100% wheat flour pizza. Also, Pečivová et al. (2013) studied pectin from apple and gum arabica from acacia tree on the quality of pizza. Using varying levels of pectin, the authors observed a pH range of 5.27 to 5.47. These observation matches the data (pH ranged from 5.14 ± 0.11 to 5.32 ± 0.13) reported in this current study.

In line with the present study, Michael et al. (2020) reported >7 log CFU/g reduction in pathogenic *E. coli* O121 population in muffins after 17 min of baking at 375°F (190.6°C). Other studies have found similar trends with different food products and pathogen. Vega (2021) reported a 3-log kill of STEC in peanut butter bars after 13 min of baking at 350°F. Furthermore, Vega documented that a lower kill in STEC can be attributed to the low a_w_ (0.811) level of peanut butter bars. Lang et al. (2017) studied the thermal inactivation of *Salmonella* Typhimurium, *Salmonella* Senftenberg, *Cronobacter sakazakii* and *E. coli* K12TG1 in whole milk powder at different water activity levels. The authors reported that these pathogens exhibited more heat resistance at lower water activity levels. When pepperoni slices were used as a source of STEC introduction, the STEC population (6.5 log CFU/g) decreased significantly compared to pizza dough and this can be attributed to direct exposure of pepperoni slices to oven heat. Michael et al. (2020) also reported the *D*- and *z*- values for *E. coli* O121 in muffin batter studied at *D*_60_, *D*_65_, and *D*_70_ as 42.0 ± 1.64, 7.5 ± 0.42, and 0.4 ± 0.03, respectively. The authors also reported the *z*- value as 5.0 ± 0.07. The difference in the *D*- and *z*-values in comparison to Michael et al. (2020) and the current study could be due to the higher muffin baking temperature. Forghani et al. (2018) studied the thermal death kinetics of Enterohemorrhagic *E. coli* (EHEC) serogroups O26, O103, O111, and O157 in wheat flour (Forghani et al., 2018). The authors reported that at 55, 60, 65, and 75°C the *D*-values for EHEC serogroups were in the range of 46.5 to 61.1, 11.2 to 16.1, 8.0 to 12.2, and 5.7 to 8.1 min, respectively. Forghani et al. (2018) reported the *z*-values for EHEC serogroups ranging from 6.4 to 13.4°C. In another study, Michael et al. (2022) determined the thermal resistance of *E. coli* O121 in muffin batter stored for extended 360 days. The authors reported the *D*-values at *D*_60_ for *E. coli* O121 in muffin batter prepared from inoculated wheat flour after 1, 30, and 90 days of storage as 47.5 ± 5.00, 41.3 ± 5.10, and 6.3 ± 1.12 min, respectively. Michael et al. also reported *D*-values at *D*_65_ for *E. coli* O121 in muffin batter after 1 and 30 days as 10.7 ± 0.03, 9.3 ± 1.67 min, respectively. The *z*-values for *E. coli* O121 in muffin batter on days 1, 30 and 90 were reported as 5.0 ± 0.19, 5.5 ± 0.26, and 8.8 ± 0.71, respectively. The comparison of *D*-values of STEC in different food matrices supports the theory that the thermal resistance of STEC increases as the water activity in food matrices declines (Forghani et al., 2018; Michael et al., 2020, 2022).

## Conclusion

This research validated that a typical traditional crust pepperoni pizza baking process with ~209°F internal temperature for 12 min will result in at least 5 log reductions in STEC population. The *D*- and *z*-values determined in this study can help researchers, regulators and bakers understand STEC’s heat resistance characteristics in pizza dough specific to the recipe used in this study. In addition, the data will be useful for bakers optimize the traditional crust pepperoni pizza baking process by effectively controlling the STEC in the event of pre-baking contamination thus protecting consumers from foodborne illness outbreaks. The food manufacturers generally use a combination of time and temperature parameters to achieve end use quality parameters and food safety. However, the major disadvantage of this step is that it is specific to the food recipe. Several intrinsic (pathogen load, a_w_, pH, fat, sugar, toppings etc.) and extrinsic (temperature, humidity etc.) factors play an important role in total process lethality. Therefore, further studies are needed to elucidate the effect of various intrinsic and extrinsic factors on the thermal resistance characteristics and total process lethality of STEC during the traditional and other types of pizza baking process.

## Data availability statement

The original contributions presented in the study are included in the article/supplementary material, further inquiries can be directed to the corresponding author.

## Author contributions

LC: designed the study, funding acquisition, conceived the experiment, interpreted the experimental data, supervision and drafted the final manuscript. AS: performed the experiment, analyzed the data, and drafted the manuscript. All authors contributed to the article and approved the submitted version.

## Funding

The authors would like to thank AIB International, Inc. (Manhattan, KS) for supporting this project. This study received funding from AIB International Inc. The funder was not involved in the study design, collection, analysis, interpretation of data, the writing of this article or the decision to submit it for publication. All authors declare no other competing interests.

## Conflict of interest

The authors declare that the research was conducted in the absence of any commercial or financial relationships that could be construed as a potential conflict of interest.

## Publisher’s note

All claims expressed in this article are solely those of the authors and do not necessarily represent those of their affiliated organizations, or those of the publisher, the editors and the reviewers. Any product that may be evaluated in this article, or claim that may be made by its manufacturer, is not guaranteed or endorsed by the publisher.

## References

[ref1] Bakerpedia (2017). Pizza. Available at: https://bakerpedia.com/processes/pizza/ (Accessed on July 01, 2022)

[ref2] Beuchat L. Komitopoulou E. Beckers H. Betts R. Bourdichon F. Fanning S.. (2013). Low-water activity foods: increased concern as vehicles of foodborne pathogens. J. Food Prot. 76, 150–172. doi: 10.4315/0362-028X.JFP-12-211, PMID: 23317872

[ref3] CDC. (2009). Multistate Outbreak of *E. coli* O157:H7 Infections Linked to Eating Raw Refrigerated, Prepackaged Cookie Dough (Final Update). Available at: https://www.cdc.gov/ecoli/2009/cookie-dough-6-30-2009.html (Accessed on July 01, 2022).

[ref4] CDC. (2016). Multistate Outbreak of Shiga Toxin-Producing *Escherichia coli* Infections Linked to Flour (Final Update). Available at: https://www.cdc.gov/ecoli/2016/o121-06-16/index.html (Accessed on July 01, 2022).

[ref5] CDC. (2018). Burden of Foodborne Illness: Questions and Answers. Available at: https://www.cdc.gov/foodborneburden/questions-and-answers.html (Accessed on July 07, 2022).

[ref6] CDC. (2021). National Shiga Toxin-Producing *Escherichia coli* (STEC) Surveillance Annual Report, 2017. Available at: https://www.cdc.gov/ecoli/surv2017/index.html (Accessed on June 20, 2022).

[ref7] CDC. (2022a). Reports of Selected *E. coli* Outbreak Investigations. Available at: https://www.cdc.gov/ecoli/outbreaks.html (Accessed on July 02, 2022).

[ref8] CDC. (2022b). Say No to Raw Dough. Available at: https://www.cdc.gov/foodsafety/communication/no-raw-dough.html (Accessed on July 08, 2022)

[ref9] Channaiah L. H. Holmgren E. S. Michael M. Sevart N. J. Milke D. Schwan C. L.. (2016). Validation of baking to control *Salmonella* Serovars in hamburger bun manufacturing, and evaluation of *Enterococcus faecium* ATCC 8459 and *Saccharomyces cerevisiae* as nonpathogenic surrogate indicators. J. Food Prot. 79, 544–552. doi: 10.4315/0362-028X.JFP-15-241, PMID: 27052857

[ref10] Channaiah L. H. Michael M. Acuff J. Lopez K. Vega D. Milliken G.. (2018). Validation of a simulated commercial frying process to control *Salmonella* in donuts. Foodborne Pathog. Dis. 15, 763–769. doi: 10.1089/fpd.2018.2440, PMID: 30407081 PMC6306667

[ref11] Channaiah L. H. Michael M. Acuff J. C. Phebus R. K. Thippareddi H. Milliken G. (2019). Evaluation of thermal inactivation parameters of *Salmonella* in whole wheat multigrain bread. Food Microbiol. 82, 334–341. doi: 10.1016/J.FM.2019.03.001, PMID: 31027791

[ref12] Crowe S. J. Bottichio L. Shade L. N. Whitney B. M. Corral N. Melius B.. (2017). Shiga toxin-producing *E. coli* infections associated with flour. New Eng. J. Med. 377, 2036–2043. doi: 10.1056/NEJMoa1615910, PMID: 29166238 PMC5792826

[ref13] Dhaliwal S. Hoffmann S. White A. Ahn J. McQueen R. B. Walter E. S. (2021). Cost of hospitalizations for leading foodborne pathogens in the United States: identification by international classification of disease coding and variation by pathogen. Foodborne Pathog. Dis. 18, 812–821. doi: 10.1089/fpd.2021.0028, PMID: 34591654

[ref14] El-Beltagi H. S. El-Senousi N. A. Ali Z. A. Omran A. A. (2017). The impact of using chickpea flour and dried carp fish powder on pizza quality. PLoS One 12:e0183657. doi: 10.1371/journal.pone.0183657, PMID: 28873098 PMC5584754

[ref15] FDA. (2017). FDA Investigated Multistate Outbreak of Shiga Toxin-Producing *E. coli* Infections Linked to Flour. Available at: https://www.fda.gov/food/outbreaks-foodborne-illness/fda-investigated-multistate-outbreak-shiga-toxin-producing-e-coli-infections-linked-flour (Accessed on June 20, 2022).

[ref16] FDA. (2018a). FDA Food Safety Modernization Act (FSMA). Available at: https://www.fda.gov/food/guidanceregulation/fsma/ (Accessed on June 27, 2022).

[ref17] FDA. (2018b). FSMA Final Rule for Preventive Controls for Human Food. Available at: https://www.fda.gov/food/guidanceregulation/fsma/ucm334115.html (Accessed on July 07, 2022).

[ref18] FDA. (2019). General Mills Recalls Five Pound Bags of Gold Medal Unbleached All Purpose Flour. Available at: https://www.fda.gov/safety/recalls-market-withdrawals-safety-alerts/general-mills-recalls-five-pound-bags-gold-medal-unbleached-all-purpose-flour (Accessed on July 01, 2022).

[ref19] FDA. (2021). Recalls, Corrections, and Removals. Available at: https://www.fda.gov/medical-devices/postmarket-requirements-devices/recalls-corrections-and-removals-devices (Accessed on July 04, 2022).

[ref20] FDA. (2022). Handling Flour Safely: What You Need to Know. Available at: https://www.fda.gov/food/buy-store-serve-safe-food/handling-flour-safely-what-you-need-know (Accessed on June 15, 2022)

[ref21] Forghani F. den Baker M. Futral A. N. Diez-Gonzalez F. (2018). Long-term survival and thermal death kinetics of enterohemorrhagic *Escherichia coli* serogroups O26, O103, O111, and O157 in wheat flour. Appl. Environ. Microbiol. 84, 1–12. doi: 10.1128/AEM.00283-18, PMID: 29678913 PMC6007106

[ref22] Forghani F. den Baker M. Liao J. Payton A. S. Futral A. N. Diez-Gonzalez F. (2019). *Salmonella* and enterohemorrhagic *Escherichia coli* serogroups O45, O121, O145 in wheat flour: effects of long-term storage and thermal treatments. Front. Microbiol. 10:323. doi: 10.3389/fmicb.2019.00323, PMID: 30853953 PMC6395439

[ref23] Gieraltowski L. Schwensohn C. Meyer S. Eikmeier D. Medus C. Sorenson A.. (2017). Notes from the field: multistate outbreak of *Escherichia coli* O157:H7 infections linked to dough mix – United States, 2016. Morb. Mortal. Wkly Rep. 66, 88–89. doi: 10.15585/mmwr.mm6603a6, PMID: 28125572 PMC5724908

[ref24] Gill A. Huszczynski G. Gauthier M. Blais B. (2014). Evaluation of eight agar media for the isolation of Shiga toxin-producing *Escherichia coli*. J. Microbiol. Methods 96, 6–11. doi: 10.1016/J.MIMET.2013.10.022, PMID: 24211606

[ref25] Gill A. McMahon T. Dussault F. Petronella N. (2020). Shiga toxin-producing *Escherichia coli* survives storage in wheat flour for two years. Food Microbiol. 87:103380. doi: 10.1016/j.fm.2019.103380, PMID: 31948621

[ref26] Harris L. J. Yada S. (2019). Flour and Cereal Grain Products: Foodborne Illness Outbreaks and Product Recalls: Tables and References. In Flour & Cereal Grains – Outbreaks and Recalls. Available at: https://ucfoodsafety.ucdavis.edu/Low_Moisture_Foods/ (Accessed on June 27, 2022).

[ref27] Kennedy S. Errecaborde K. M. Hueston W. (2014). General Overview of the Food Safety Modernization Act. Food Policy Research Center, University of Minnesota, St. Paul, MN.

[ref28] Lang E. Chemlal L. Molina P. Guyot S. Alvarez-Martin P. Perrier-Corneta J.. (2017). Modeling the heat inactivation of foodborne pathogens in milk powder: high relevance of the substrate water activity. Food Res. Int. 99, 577–585. doi: 10.1016/J.FOODRES.2017.06.028, PMID: 28784519

[ref29] Marler B. (2022). Publisher’s platform: frozen pizzas from the Nestlé Buitoni brand Fraîch’Up Likely Have Sickened Several Hundred. Food Safety News. Available at: https://www.foodsafetynews.com/2022/05/publishers-platform-frozen-pizzas-from-the-nestle-buitoni-brand-fraichup-likely-have-sickened-several-hundred/?utm_source=Food+Safety+News&utm_campaign=0c14266464-RSS_EMAIL_CAMPAIGN&utm_medium=email&utm_term=0_f46cc10150-0c14266464-40613596 (Accessed June 13, 2022).

[ref30] Michael M. Acuff J. C. Lopez K. Vega D. Phebus R. K. Thippareddi H.. (2020). Comparison of survival and heat resistance of *Escherichia coli O121* and *Salmonella* in muffins. Int. J. Food Microbiol. 317:108422. doi: 10.1016/J.IJFOODMICRO.2019.108422, PMID: 31756646

[ref31] Michael M. Acuff J. C. Vega D. Sekhon A. S. Channaiah L. H. Phebus R. K. (2022). Survivability and thermal resistance of *salmonella* and *Escherichia coli* O121 in wheat flour during extended storage of 360 days. Int. J. Food Microbiol. 362:109495. doi: 10.1016/J.IJFOODMICRO.2021.109495, PMID: 34872756

[ref32] Morton V. Cheng J. M. Sharma D. Kearney A. (2017). An outbreak of Shiga toxin-producing *Escherichia coli* O121 infections associated with flour-Canada, 2016-2017. Can. Commun. Dis. Rep. 43, 154–155. doi: 10.14745/ccdr.v43i78a03, PMID: 29770083 PMC5864315

[ref33] Pečivová P. Dula T. Hrabě J. (2013). The influence of pectin from apple and gum Arabic from acacia tree on the quality of pizza. Int. J. Food Prop. 16, 1417–1428. doi: 10.1080/10942912.2011.587931

[ref34] Singh P. Wani A. A. Goyal G. K. (2012). Shelf-life extension of fresh ready-to-bake pizza by the application of modified atmosphere packaging. Food Bioprocess Technol. 5, 1028–1037. doi: 10.1007/S11947-010-0447-9/TABLES/3

[ref35] Tack D. M. Kisselburgh H. M. Richardson L. C. Geissler A. Griffin P. M. Payne D. C.. (2021). Shiga toxin-producing *Escherichia coli* outbreaks in the United States, 2010–2017. Microorganisms. 9:1529. doi: 10.3390/microorganisms9071529, PMID: 34361964 PMC8307841

[ref36] Unger P. Channaiah L. H. Singh A. Sekhon A. S. Babb M. Yang Y.. (2021). Validation of brownie baking step for controlling *Salmonella* and *Listeria monocytogenes*. Food Sci. Nutr. 9, 1574–1583. doi: 10.1002/FSN3.2132, PMID: 33747470 PMC7958536

[ref37] USDA. (2012). Introduction to the Microbiology of the Food Poisoning - Small Plant News Guidebook Series. Available at: https://www.fsis.usda.gov/shared/PDF/SPN_Guidebook_Microbiology.pdf (Accessed on March 12, 2022)

[ref38] Vega D. (2021). Food Safety Interventions in the Bakery Industry: Microbial Safety from Wheat Milling to Finished Baked Products. [Dissertation]. [Manhattan (KS)] Kansas State University. Available at: https://krex.k-state.edu/dspace/bitstream/handle/2097/41717/DanielVega2021.pdf?sequence=3&isAllowed=y (Accessed May 26, 2022).

[ref39] Woodruff J. (2019). Bakery Industry Analysis. Available at: https://smallbusiness.chron.com/bakery-industry-analysis-64831.html (Accessed on July 04, 2022).

